# Novel *Rickettsia* Species Infecting Dogs, United States

**DOI:** 10.3201/eid2612.200272

**Published:** 2020-12

**Authors:** James M. Wilson, Edward B. Breitschwerdt, Nicholas B. Juhasz, Henry S. Marr, Joao Felipe de Brito Galvão, Carmela L. Pratt, Barbara A. Qurollo

**Affiliations:** North Carolina State University College of Veterinary Medicine, Raleigh, NC, USA (J.M. Wilson, E.B. Breitschwerdt, N.B. Juhasz, H.S. Marr, B.A. Qurollo);; VCA Arboretum View Animal Hospital, Downers Grove, Illinois, USA (J.F. de Brito Galvão);; Oklahoma Veterinary Specialists, Tulsa Oklahoma, USA (C.L. Pratt)

**Keywords:** Spotted-fever group Rickettsia, tickborne diseases, vector-borne diseases, parasites, bacteria, Rickettsia, zoonoses, United States, dogs

## Abstract

In 2018 and 2019, spotted fever was suspected in 3 dogs in 3 US states. The dogs had fever and hematological abnormalities; blood samples were *Rickettsia* seroreactive. Identical *Rickettsia* DNA sequences were amplified from the samples. Multilocus phylogenetic analysis showed the dogs were infected with a novel *Rickettsia* species related to human *Rickettsia* pathogens.

In the United States, tickborne *Rickettsia parkeri*, *R. philipii* (*Rickettsia* 364D), and *R. rickettsii*, causative agents of Rocky Mountain spotted fever (RMSF), are well-documented human spotted fever group (SFG) rickettsioses (*1*). *R. rickettsii* is the only known cause of SFG rickettsioses in dogs (*2*). The extent to which other SFG *Rickettsia* are pathogenic in dogs is unclear; however, SFG *Rickettsia* seroprevalence is high among dogs in the United States and Mexico (*3*,*4*). The increased *R. rickettsii* seroprevalence in humans in the United States during the past decade has been attributed to SFG *Rickettsia* cross-reactivity (*1*,*5*).

We report 3 dogs with febrile illness located in different US states. Samples from the dogs were *R. rickettsii* seroreactive. Identical *Rickettsia* DNA gene sequences were obtained from each dog’s blood specimen and used to investigate *Rickettsia* spp.

## The Cases

On May 15, 2018, a 10-year-old male neutered mixed breed dog (case 1) from Tennessee was examined by a veterinarian for lethargy and hyporexia. The owner reported removing a tick (species unknown) within the previous 2 weeks. On physical examination, the dog had fever (39.8°C) and possible hepatomegaly. Radiographic imaging results were unremarkable. Thrombocytopenia was the only abnormality noted on complete blood count (CBC). Serum biochemistry panel (SBP) abnormalities included hyperglobulinemia, increased serum alkaline phosphatase activity, hypoglycemia, and hyponatremia (Table 1). Results of urine dipstick and sediment examination were unremarkable. The dog’s samples were *R. rickettsii* seroreactive and PCR positive for *Rickettsia* (Table 2). Clinical abnormalities resolved after treatment with doxycycline, and the dog remained healthy during the 1-year follow-up period.

**Table 1:** Findings from physical examination, laboratory results, treatment regimens for 3 dogs infected with a novel *Rickettsia* species, United States*

Examination and treatment	Case 1	Case 2	Case 3
Physical examination	Febrile (39.8°C); lethargy; +/− hepatomegaly	Febrile (40.1°C); lethargy; dehydration; joint effusion (elbow, carpus, and tarsus); arthropathy; shifting leg lameness	Febrile (39.8°C); lethargy; abdominal pain
CBC	Platelets 141 × 10^3^ cells/µL (RI 200–500 × 10^3^ cells/µL)	Platelets 139 × 10^3^ cells/µL (RI 170–400 × 10^3^ cells/µL)	Platelets 60 × 10^3^ cells/µL (RI 125–500 × 10^3^ cells/µL); Hct 35.2% (RI 36%–55%); 2 d later platelets 25 × 10^3^ cells/µL and Hct 26.8%
SBP	Globulins 4.5 g/dL (RI 2.1–4.4 g/dL); ALP 177 U/L (RI 11–140 U/L); glucose 73 mg/dL (RI 75–125 mg/dL); sodium 136.5 mmol/L (RI 143–153 mmol/L)	Albumin 2.2 g/dL (RI 2.7–4.4 g/dL); ALT 1,158 U/L (RI 12–118 U/L); ALP 1,702 U/L (RI 5–131 U/L); cholesterol 352 mg/dL (RI 92–324 mg/dL); calcium 8.4 mg/dL (RI 8.9–11.4 mg/dL)	Albumin 1.0 mg/dL (RI 2.5–4.3 mg/dL); calcium 8.4 mg/dL (RI 8.9–11.4 mg/dL); BUN 35 (35, RI 7–28 mg/dL)
Urinalysis	USG 1.007	Microalbuminuria 3.1 (RI <2.5 mg/dL)	USG 1.033; 3+ proteinuria; UPC 14.7 (RI 0.00–1.00)
Treatment regimen	Doxycycline (6 mg/kg every 12 h for 21 d)	Doxycycline (7 mg/kg every 12h for 28 d); prednisone (1 mg/kg every 12 h for 9 mo with gradual taper); omeprazole (1.4 mg/kg every 12h for 9 mo); ondansetron (0.5 mg/kg every 12h for 15 d); and metronidazole (17 mg/kg every 12 h for 15 d)	Doxycycline (7.5 mg/kg q12 h for 40 d); prednisone (1 mg/kg every 12 h for 14 d, then 0.5mg/kg every 12 h for 6 d, then every 24 h for 22 d until death), mycophenolate (12.5 mg/kg every 12 h for 22 d until death)

*ALP, alkaline phosphatase activity; ALT, alanine amino transferase activity; BUN, blood urea nitrogen; CBC, complete blood count; Hct, hematocrit; RI, reference interval; SBP, serum biochemistry panel; UA, urinalysis; UPC, urine protein/creatinine ratio; USG, urine specific gravity.

**Table 2:** Canine vector-borne disease diagnostic results for blood and serum samples from 3 dogs infected with a novel *Rickettsia* species*

Sample dates	CVBD panel†	IFA‡	*Rickettsia* PCR§									
			23S-5S ITS	*htrA* (17kDa)	*mmpA*-*purC* ITS	*gltA* region				*ompA* region		
						1	2	3		1	2	3
Case 1												
2018 May 5¶	–	1:512	+	+	+	+	+	+		+	+	–**
Case 2												
2019 May 8¶	–	1:256	+	+	+	+	NA††	+		+	+	NA††
2019 May 15	–	1:8,192	–	NA	NA	NA	NA	NA		NA	NA	NA
2019 May 28	–	1:1,024	–	NA	NA	NA	NA	NA		NA	NA	NA
2019 Jul 16	NA	1:2,048	NA	NA	NA	NA	NA	NA		NA	NA	NA
2019 Oct 2	–	1:2,048	–	NA	NA	NA	NA	NA		NA	NA	NA
2019 Nov 12	–	1:2,048	–	NA	NA	NA	NA	NA		NA	NA	NA
Case 3												
2019 Aug 28¶‡‡	–	1:1,024	+	+	+	+	+	+		+	+	+
2019 Sep 10	NA	1:8,192	–	NA	NA	NA	NA	NA		NA	NA	NA
*CVBD, canine vectorborne disease; IFA, immunofluorescence assay; NA, not applicable; +, positive; –, negative. †Panel includes IFA serology for *Babesia canis vogeli*, *B. gibsoni*, *Bartonella henselae*, *B. koehlerae*, *B. vinsonii berkhoffii*, and *Ehrlichia canis*; point-of-care ELISA serology test SNAP 4DX Plus for *Dirofilaria immitis* antigen and antibodies against *Anaplasma phagocytophilum*, *A. platys*, *Borrelia burgdorferi*, *Ehrlichia canis*, and *E. ewingii*; and PCR for *Anaplasma*, *Babesia*, *Bartonella*, *Ehrlichia*, hemotropic *Mycoplasma, Neoehrlichia*, and *Neorickettsia.* ‡IFA results are reported as reciprocal titers. All samples were positive for *R. rickettsii.* §PCR assay gene targets 23S-5S ITS, *htrA* (17 kDa), *mmpA*-*purC* ITS, *gltA*, and *ompA*. ¶Sample tested before doxycycline treatment administered. **The PCR was negative despite repeated attempts. *ompA* region 3 PCR assay was designed to bridge *ompA* regions 1 and 2 to obtain an additional 281 bps. The total amplicon size of *ompA* region 3 is 533 bp (Appendix Table). DNA from case 1 was >1 year old when retrospective PCRs were performed. Poor DNA quality might have prevented amplification of the larger amplicon. ††PCR assays were not performed due to depleted blood sample for DNA extraction and testing. ‡‡GenBank accession nos. for sequences from case 3: 23S-5S ITS, MT050448; *htrA *(17 kDa), MT050446;* mmpA*-*purC* ITS, MT066187; *gltA*, MT050445; and *ompA*, MT050447.												

On May 8, 2019, a 9-year-old male neutered Boston terrier (case 2) from Illinois was examined by a veterinarian for lethargy, difficulty walking, and painful elbows. Clinical signs developed 3 days after returning from a tick-infested area in Arkansas. Abnormalities noted on physical examination included fever (40.1°C), dehydration, joint effusion, elbow pain, and shifting leg lameness. Thrombocytopenia and mild leukocytosis were the only CBC abnormalities (Table 1). SBP abnormalities included hypoalbuminemia, increased alanine amino transferase activity, alkaline phosphatase activity, hypercholesterolemia, and hypocalcemia (Table 1). Mild microalbuminuria was noted. Neutrophilic inflammation was documented by synovial fluid cytology in the right and left stifle joints, right tarsus, and left elbow joint. The left carpus contained moderate, chronic inflammation with very rare extracellular cocci; however, culture resulted in no bacterial growth. The dog experienced cardiorespiratory arrest during sedated arthrocentesis but recovered after CPR and sedative reversal. Thoracic radiographs were unremarkable. The dog’s samples were *R. rickettsii* seroreactive and PCR–positive for *Rickettsia* and convalescent titers demonstrated 4-fold seroconversion (Table 2). Most clinical abnormalities resolved after administration of doxycycline to treat rickettsiosis, prednisone to treat potential immune-mediated component, omeprazole to prevent gastric ulcers, and metronidazole to treat assumed dysbiosis. All SBP changes resolved within 5 months of treatment and the dog remained healthy during the 5-month follow-up.

On August 28, 2019, a 9-year-old male neutered terrier mixed-breed (case 3) from Oklahoma was examined by a veterinarian for lethargy, hyporexia, and polydipsia. Physical examination revealed fever (39.8°C) and palpable abdominal tenderness. CBC abnormalities included a normocytic normochromic anemia and thrombocytopenia. SBP abnormalities included hypoproteinemia, hypocalcemia, and mild azotemia. A protein-losing nephropathy (PLN) was documented by urine dipstick and protein/creatinine ratio. Blood samples were *R. rickettsii* seroreactive and *Rickettsia* PCR positive, and convalescent titers demonstrated 4-fold seroconversion (Table 2). 

The year before, in August 2018, the dog described in case 3 was examined by a veterinarian for lethargy after tick attachment. At that time, fever (39.7°C), anemia, thrombocytopenia, hyperbilirubinemia, and hypoproteinemia were documented. IFA serology tests performed by 1 diagnostic laboratory showed samples were *R. rickettsii* seroreactive (1:320) but seronegative for *Anplasma* spp., *Borrelia burdorferi*, and *Ehrlichia* spp. by SNAP 4Dx Plus (IDEXX Laboratories, https://www.idexx.com). Doxycycline and immunosuppressive doses of prednisone were administered concurrently for RMSF and potential immune-mediated disease. Clinical and hematologic abnormalities resolved, and treatment was transitioned from prednisone to cyclosporine due to adverse side effects. Cyclosporine was discontinued in January 2019 and serial monthly CBCs remained normal through March 2019. When rechecked on August 9, 2019, for joint pain, hematocrit and platelet count were normal, but hypoproteinemia, hypoalbuminemia, and hypocalcemia were detected. By August 30, 2019, the dog’s anemia and thrombocytopenia worsened, despite treatment with doxycycline and prednisone. Marked abdominal effusion was documented by abdominal ultrasound, without evidence of an intra-abdominal mass. Prednisone and mycophenolate were administered for presumptive immune-mediated thrombocytopenia, and within 3 weeks, the platelet count normalized and titers increased by 4-fold. Despite medical therapy for PLN, nephrotic syndrome developed, and the dog was euthanized.

We obtained identical *Rickettsia* DNA gene sequences from each dog’s blood specimen. We confirmed novel *Rickettsia* sp. by PCR targeting 3 genes (*gltA*, *htrA*, and *ompA*) and 2 intergenic spacer regions (23S-5S and *mmpA-purC*) (Table 2). *Rickettsia* amplicons were 100% identical among the 3 dogs. We amplified a larger region of the *ompA* and *gltA* genes by using 3 different quantitative PCRs from case 3. We submitted sequences from this dog’s serum samples to GenBank (accession nos. MT050445–8 and MT066187). We also used the *Rickettsia* sequences from case 3 to generate a phylogenetic tree (Table 2) based on concatenated novel *Rickettsia* sp. DNA sequences and reference *Rickettsia* spp. We generated the phylogenetic tree by using the maximum-likelihood method based on the Tamura-Nei model (Figure) (*6*,*7*). Multilocus phylogenetic analysis placed the novel *Rickettsia* sp. in a clade among SFG *Rickettsia* between the human pathogens *R. heilongjiangensis* and *R. massiliae*. We attempted cell culture isolation of the *Rickettsia* sp. from whole blood but were unsuccessful (Appendix).

**Figure:**
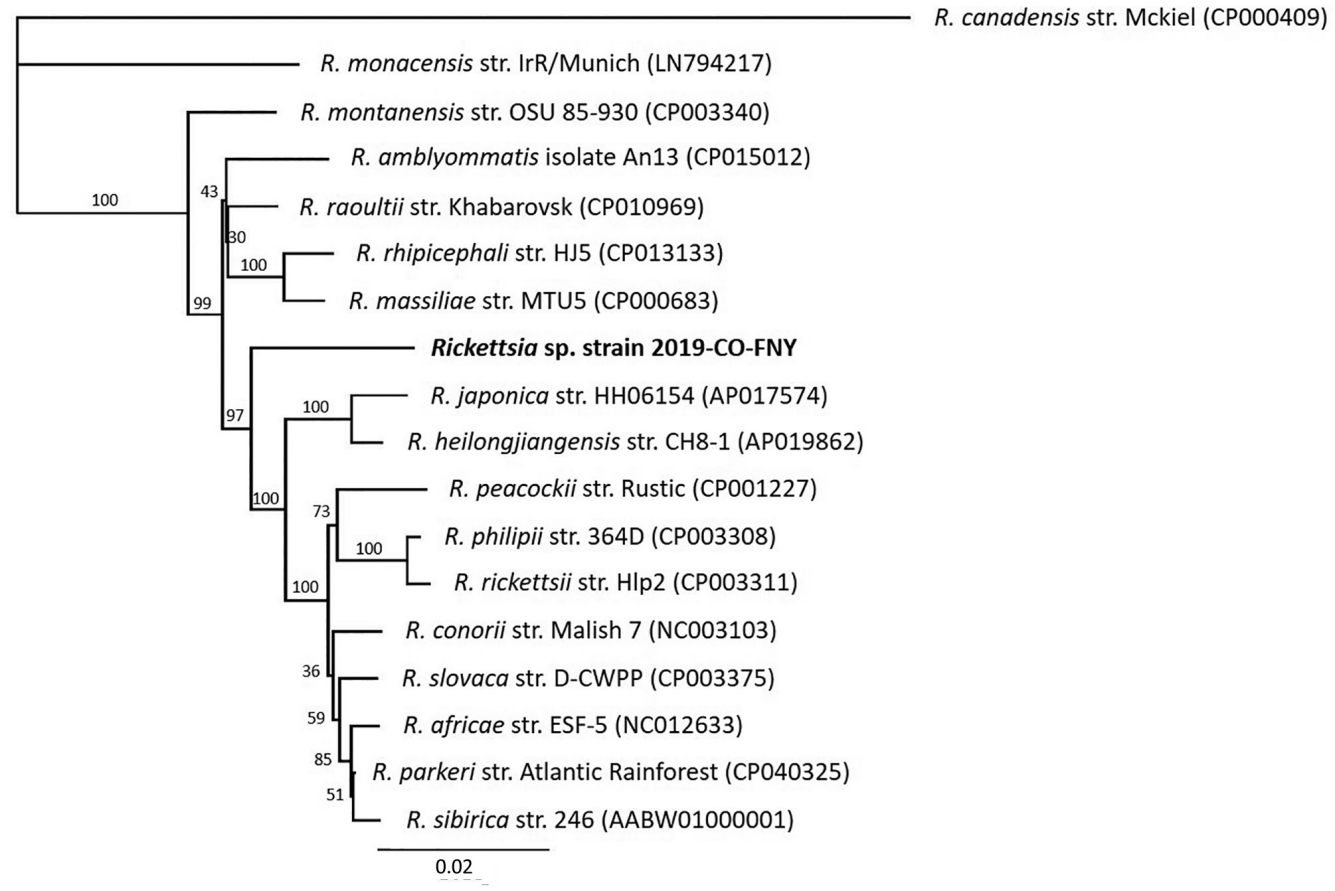
Multilocus phylogenetic tree of *Rickettsia* spp. obtained from a dog with Rocky Mountain spotted fever–type symptoms in 2019 (bold) compared with reference sequences. We noted 3 dogs with RSMF symptoms. *Rickettsia* DNA were identical among all 3 cases; however, complete sequences from all 5 regions were obtained only from case 3, which we used to represent the novel *Rickettsia* species strain 2019-CO-FNY. We used 2,576 nucleotides concatenated from regions within 3 genes (*gltA*, *htrA*, and *ompA*) and 2 intergenic spacer regions (23S-5S and *mmpA-purC*). We used the maximum-likelihood method and Tamura-Nei model (*6*,*7*) optimized for branch length, topology, and substitution rate to assemble the tree by using the PhyML 3.3.20180621 plugin in Geneious Prime 11.0.0+7 (https://www.geneious.com). Numbers at nodes indicate bootstrap percentages obtained from 1,000 resamplings. Numbers in parentheses are GenBank accession numbers. The tree is drawn to scale. Scale bar indicated the number of nucleotide substitutions per site.

## Conclusions

We report similar illnesses among 3 dogs from different US states associated with tick exposures occurring in summer months. All 3 cases demonstrated fever, lethargy, and thrombocytopenia, abnormalities commonly associated with RMSF. Case 1 had a typical acute onset fever and rapidly responded to treatment with doxycycline; case 2 had a neutrophilic polyarthritis, which has been associated with RMSF in dogs (*8*). Case 3 was examined for acute onset febrile illness 1 year before the novel *Rickettsia* sp. infection was documented; *Rickettsia* IFA seroreactivity was documented on both occasions. This dog likely had an unidentified, concurrent disease process that contributed to PLN.

The cases were geographically distributed among 4 states; the dogs resided in Illinois, Oklahoma, and Tennessee, but the dog from Illinois had traveled to a tick-infested area of Arkansas. The tick species were not identified, but ticks common to these states include *Amblyomma americanum, Dermacentor variabilis,* and *Rhipicephalus sanguineus* sensu lato, all of which are known to transmit *Rickettsia* (*3*). *Haemophysalis longicornis*, an invasive tick species recently confirmed in the United States, including in Tennessee and Arkansas, should be considered a potential vector for *Rickettsia* spp. (*9*,*10*).

Based on serologic cross-reactivity, presence of *ompA*, and phylogenetic tree analysis, the new *Rickettsia* sp. is an SFG *Rickettsia*, phylogenetically related to human pathogenic *R. heilongjiangensis* and *R. massiliae*, with only 95% identity to each (*11*,*12*). Thus, we report a previously unknown and unique *Rickettsia* sp. with clinical significance for dogs and potentially humans. Because this novel *Rickettsia* cross-reacts with *R. rickettsia* on IFA, it could be underdiagnosed and more geographically widespread. Studies aimed at identifying the tick vector, potential animal reservoirs, and prevalence are ongoing. These 3 canine rickettsioses cases underscore the value of dogs as sentinels for emerging tickborne pathogens (*13*,*14*).

Appendix Additional methods used to investigate cases of a novel *Rickettsia* sp. among 3 dogs, United States.
