# Supplementary material for: Novel Rickettsia Species Infecting Dogs, United States
Source: Emerg Infect Dis. 2020 Dec;26(12):3011–5. doi: 10.3201/eid2612.200272 (PMC7706976; doi:10.3201/eid2612.200272)
Supplement: Appendix — Additional methods used to investigate cases of a novel Rickettsia sp. among 3 dogs, United States. [file 20-0272-Techapp-s1.pdf]

Article DOI: https://doi.org/10.3201/eid2612.200272

Novel *Rickettsia* Species Infecting Dogs, United States

Appendix

Molecular Diagnostic Testing

EDTA-anticoagulated whole blood was submitted to the North Carolina State College of Veterinary Medicine, Vector-Borne Disease Diagnostic Laboratory (VBDDL) for vector-borne disease PCR testing. DNA was extracted from 200 µL aliquots of whole blood by using a QIAsymphony SP robot (QIAGEN, https://www.qiagen.com) and QIAsymphony DNA Mini Kit (QIAGEN). All sets of extractions included negative extraction controls of molecular-grade water. DNA was stored at –20°C before PCR testing. The absence of PCR inhibitors was demonstrated by an internal control quantitative PCR (qPCR) designed to amplify the host glyceraldehyde-3-phosphate dehydrogenase (GAPDH) gene (*1*). Samples were tested for *Rickettsia* spp. DNA by using *Rickettsia*-specific PCR assays targeting 6 different DNA regions (Appendix Table). All PCR assays were run with negative molecular-grade water, a negative control of known uninfected canine DNA, and a DNA–positive control extracted from *R. conorii* and *R. rickettsia* cultures. Amplification assays were performed in CFX96 Real-Time Detection System combined with C1000 Thermal Cycler (Bio-Rad, https://www.bio-rad.com) for qPCR and an Eppendorf Mastercycler EPgradient with aluminum block for cPCR. Amplification reactions contained 12.5 µL SYBR Green Supermix (Bio-Rad) for qPCRs or MyTaq HS Mix (2×) (Bioline, https://www.bioline.com) for cPCRs, 5 µL of DNA template, primers at final concentration of 0.4 µMol and molecular-grade water to a final volume of 25 µL. We provide details on PCR reaction conditions and *Rickettsia* gene targets and references for PCRs we did not develop (*2*–*6*) (Appendix Table).

Rickettsia rickettsii IFA Serologic Testing

Serial, 2-fold dilutions of canine serum samples were made in PBS solution containing 0.05% Tween 20, 0.5% non-fat dry milk, and 1% normal goat serum (GIBCO, Fisher Scientific, https://www.fishersci.com) before adding 8–10 µL to slide wells prepared with *R. rickettsia,* originally isolated from a naturally infected dog, cultured in DH82 cells. Slides containing serial diluted canine serum was incubated in a humidified chamber at 37°C for 30 minutes and washed in PBS at room temperature at 300 rpm for 30 minutes. Slides were then air-dried before adding 8–10 µL of a 0.01 mg/mL solution of fluorescein isothiocyanate (FITC) goat anti-dog immunoglobulin G (H&L) conjugate (Sigma, https://www.sigmaaldrich.com) to each well. Slides were then incubated in a humidified chamber at 37°C for 30 min before being washed in ≈400 mL PBS at room temperature, in the dark, at 300 rpm for 20 min. Slides were washed for an additional 20 min after adding 4–5 drops of a Tween-20. Slides were then rinsed with deionized water and dried in the dark before adding a coverslip with antifading mounting medium, Vectashield (Vector Laboratories, https://vectorlabs.com). Slides were evaluated by using a ZEISS Axio Lab.A1 fluorescence microscope with exciter and barrier filters (Carl Zeiss Microscopy, https://www.zeiss.com) under ×400 magnification. Each slide contained canine seroreactive *R. rickettsia* positive control serum and canine nonreactive negative control serum. Canine serum samples were screened at 1:16, 1:32 and 1:64 dilutions, and all serum samples reactive at a titer of 1:64 were repeated and diluted to an endpoint titer of 1:8,192. To avoid confusion with possible nonspecific binding found at low dilutions, a cutoff titer of ≥1:64 was used to define a seroreactive titer.

Rickettsia Culture

For cases 1 and 2, we attempted to grow the new *Rickettsia* sp. in DH82 canine cells with a modified protocol (*7*). EDTA-anticoagulated whole blood (0.5 mL) was added to a 95% confluent monolayer of DH82 canine cells in a T-10 flask with 1 mL of media (RPMI/10% FBS). The flask was rocked slowly at room temperature for 1 hour before adding 3 mL of fresh media (RPMI/10% FBS) and placing in a 37°C incubator with 5% CO_2. After 12 hours of incubation, all nonadherent content was added to a T-25 flask of 85% confluent DH82 cells. Fresh media was added to both flasks and placed in a 37°C incubator with 5% CO_2. After 12

hours of incubation, the contents of both T-10 and T-25 flasks were combined into a T-75 flask, and fresh media was added to the used T-10 and T-25 flasks. Content from all flasks were incubated for 2 weeks and periodically tested by *Rickettsia* qPCR and Diff-Quick staining (RAL Diagnostics, https://www.ral-diagnostics.fr) to identify *Rickettsia* organisms.

Sequencing and Phylogenetic Analyses

Nucleotide sequencing of amplicons was performed by Genewiz, Inc. (https://www.genewiz.com) by using both forward and reverse primers for each DNA target. DNA sequences were analyzed by using the BLAST search algorithm and the NCBI nucleotide database and sequence alignments were performed by using Geneious Prime (https://www.geneious.com) for Java version 11.0.2+7. Sequences were deposited into NCBI nucleotide database. A total of 2,576 aligned nucleotides from regions within 3 genes (*ompA*, *gltA*, and 17 kDa) and 2 intergenic spacers (23S-5S and *mmpA-purC*), were concatenated and a multilocus phylogenetic tree was assembled by using maximum-likelihood method and Tamura-Nei model through PhyML 3.3.20180621and Geneious Prime software (*8*,*9*).

References

1. Birkenheuer AJ, Levy MG, Breitschwerdt EB. Development and evaluation of a seminested PCR for detection and differentiation of *Babesia gibsoni* (Asian genotype) and *B. canis* DNA in canine blood samples. J Clin Microbiol. 2003;41:4172–7. PubMed https://doi.org/10.1128/JCM.41.9.4172-4177.2003

2. Tyrrell JD, Qurollo BA, Tornquist SJ, Schlaich KG, Kelsey J, Chandrashekar R, et al. Molecular identification of vector-borne organisms in *Ehrlichia* seropositive Nicaraguan horses and first report of *Rickettsia felis* infection in the horse. Acta Trop. 2019;200:105170. PubMed https://doi.org/10.1016/j.actatropica.2019.105170

3. Kidd L, Maggi R, Diniz PP, Hegarty B, Tucker M, Breitschwerdt E. Evaluation of conventional and real-time PCR assays for detection and differentiation of spotted fever group rickettsia in dog blood. Vet Microbiol. 2008;129:294–303. PubMed https://doi.org/10.1016/j.vetmic.2007.11.035

4. Eremeeva ME, Dasch GA, Silverman DJ. Evaluation of a PCR assay for quantitation of *Rickettsia rickettsii* and closely related spotted fever group rickettsiae. J Clin Microbiol. 2003;41:5466–72. PubMed https://doi.org/10.1128/JCM.41.12.5466-5472.2003

5. Peckle M, Luz HR, Labruna MB, Serpa MCA, Lima S, Maturano R, et al. Multi-locus phylogenetic analysis groups the New World bacterium *Rickettsia* sp. strain ApPR with the Old World species *R. africae*; proposal of “*Candidatus* Rickettsia paranaensis”. Ticks Tick Borne Dis. 2019;10:101261. PubMed https://doi.org/10.1016/j.ttbdis.2019.07.005

6. Labruna MB, Whitworth T, Horta MC, Bouyer DH, McBride JW, Pinter A, et al. *Rickettsia* species infecting *Amblyomma cooperi* ticks from an area in the state of São Paulo, Brazil, where Brazilian spotted fever is endemic. J Clin Microbiol. 2004;42:90–8. PubMed https://doi.org/10.1128/JCM.42.1.90-98.2004

7. Luce-Fedrow A, Wright C, Gaff HD, Sonenshine DE, Hynes WL, Richards AL. In vitro propagation of *Candidatus* Rickettsia andeanae isolated from *Amblyomma maculatum.* FEMS Immunol Med Microbiol. 2012;64:74–81. PubMed https://doi.org/10.1111/j.1574-695X.2011.00905.x

8. Tamura K, Nei M. Estimation of the number of nucleotide substitutions in the control region of mitochondrial DNA in humans and chimpanzees. Mol Biol Evol. 1993;10:512–26. PubMed https://doi.org/10.1093/oxfordjournals.molbev.a040023

9. Guindon S, Dufayard JF, Lefort V, Anisimova M, Hordijk W, Gascuel O. New algorithms and methods to estimate maximum-likelihood phylogenies: assessing the performance of PhyML 3.0. Syst Biol. 2010;59:307–21. PubMed https://doi.org/10.1093/sysbio/syq010

Appendix Table. Quantitative and conventional PCR assay conditions, PCR targets, primers, and associated references used in an investigation of novel *Rickettsia* in dogs, USA

Gene target (*reference*)	Primers	Primer Sequence (5′–3′)	Primer, µMol	PCR product, bp	Polymerase A/D	D	A	E	No. DAE cycles	Final E
23s-5s ITS (*2*)	Rick23-5_F2	AGC TCG ATT GAT TTA CTT TGC TG	0.4	215	98°C, 180 s	98°C, 15 s	62°C, 15 s	72°C, 20 s	40	NA
	Rick23-5_R	CCA CCA AGC TAG CAA TAC AAA	0.4							
OmpA_1† (*3*)	OmpA 107F	GCT TTA TTC ACC ACC TCA AC	0.4	137	95°C, 120 s	95°C, 10 s	58°C, 10 s	72°C, 10 s	50	72°C, 30 s
	OmpA 299R	TRA TCA CCA CCG TAA GTA AAT	0.4							
OmpA_2 (*4*)	Rr190.547F	CCT GCC GAT AAT TAT ACA GGT TTA	0.4	115	98°C, 180 s	98°C, 15 s	60°C, 15 s	72°C, 20 s	40	
	Rr190.701R	GTT CCG TTA ATG GCA GCAT	0.4							
OmpA_3 (*4*)	OmpA 107F	GCT TTA TTC ACC ACC TCA AC	0.4	533	95°C, 120 s	95°C, 10 s	60°C, 10 s	72°C, 10 s	50	72°C, 30 s
	Rr190.701R	GTT CCG TTA ATG GCA GCAT	0.4							
17kDa	Rck 17kDa F	GCG CAT TAC TTG GTT CTCA A	0.4	173	98°C, 180 s	98°C, 15 s	60°C, 15 s	72°C, 15 s	40	NA
	Rck 17kDa R	GTA GAA TGG CGT AAT CCG GA	0.4							
mmpA-purC ITS† (*5*)	mppA F	CAA ATG GCT CAA GAG AAA AA	0.4	507	95°C, 120 s	95°C, 15 s	60°C, 15 s	72°C, 30 s	40	72°C, 60 s
	mppA R	TTT TCA ATG CCG ATC ATT TC	0.4							
GltA_1†	GltA F	TGC GGA AGC CGA TTG CTT TAC	0.4	847	95°C, 120 s	95°C, 10 s	58°C, 10 s	72°C, 10 s	50	72°C, 30 s
	GltA R	AGC TGC CCG AGT TCC TTT AAT AC	0.4							
GltA_2	GltA F2	CAG TAC TTA AAG AAA CGT GCA AAG	0.4	222	98°C, 180 s	98°C, 15 s	57°C, 15 s	72°C, 20 s	40	NA
	CS-6	AGG GTC TTC GTG CAT TTC TT	0.4							
GltA_3 (*6*)	CS-5	GAG AGA AAA TTA TAT ATC CAA ATG TTG AT	0.4	99	98°C, 180 s	98°C, 15 s	57°C, 15 s	72°C, 20 s	40	NA
	CS-6	AGG GTC TTC GTG CAT TTC TT	0.4							

*A, annealing; A/D, activation/denaturization; D, denaturization; DAE, denaturation-annealing-extension; E, extension.
†Conventional PCR.
